# Supplementary material for: Pre-Bleaching Coral Microbiome Is Enriched in Beneficial Taxa and Functions
Source: Microorganisms. 2024 May 16;12(5):1005. doi: 10.3390/microorganisms12051005 (PMC11123844; doi:10.3390/microorganisms12051005)
Supplement: Supplementary file 1 [file microorganisms-12-01005-s001.zip › microorganisms-2921353-supplementary.pdf]

Supplemental Material

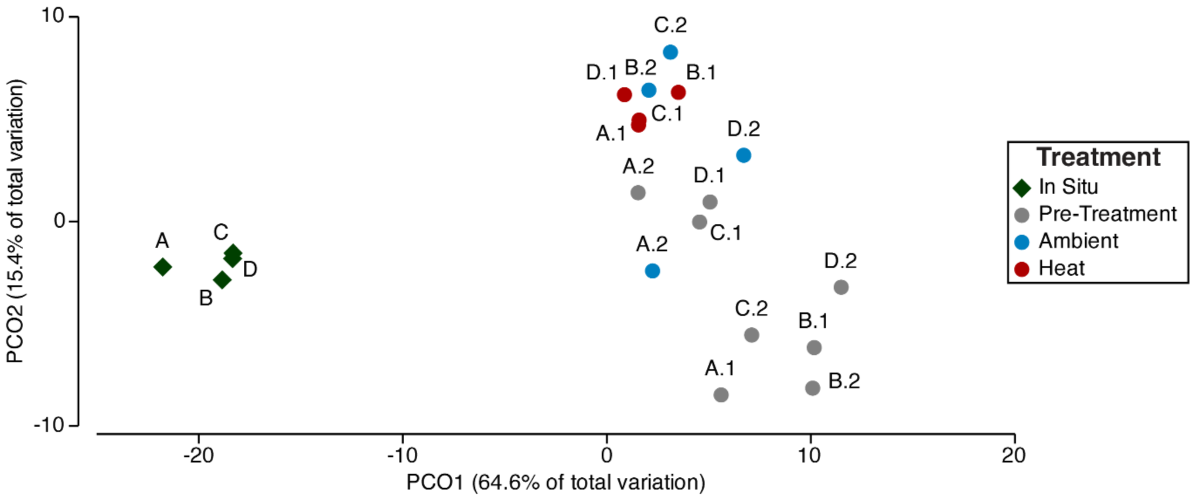

Figure S1. Principal coordinate analyses of the metagenomes associated with the coral SML microbiome of *P. strigosa* exposed to different temperature treatments. The microbiomes of four coral colonies (A, B, C, D) were sampled in their natural environment before the coral colonies were removed and replicated into two fragments (coral nubbins) each (A.1, A.2, B.1, B.2, C.1, C.2, D.1, D.2). After an acclimation period of two weeks, the microbiomes of the coral nubbins were sampled before (pre-treatment) and after being exposed to ambient and heat treatments for one week. The analysis was based on a Bray-Curtis similarity matrix of the relative abundances of the bacterial genera.

Table S1. Metagenomic sequences coverage from each sample available on the MG-RAST server.

Supplemental Table 1. Metagenomic sequences coverage in MG-RAST server

Metagenome name	Coral colony ID	Coral nubbin ID	Treatment	Total number of sequences
IR1AA-1-Inner-Heat-R1-21Jun2017	IR-1	IR-1.1	Heat	683,893
IR1AA-1-Inner-Pre-R3-15Jun2017	IR-1	IR-1.1	Pre-treatment	368,182
IR1AA-4-Inner-Ambient-R3-21Jun2017	IR-1	IR-1.2	Ambient	356,426
IR1AA-4-Inner-Pre-R2-15Jun2017	IR-1	IR-1.2	Pre-treatment	453,083
IR3LL1-3-Inner-Heat-R3-21Jun2017	IR-2	IR-2.1	Heat	498,213
IR3LL1-3-Inner-Pre-R4-15Jun2017	IR-2	IR-2.1	Pre-treatment	460,170
IR3LL1-4-Inner-Ambient-R2-21Jun2017	IR-2	IR-2.2	Ambient	1,009,994
IR3LL1-4-Inner-Pre-R3-15Jun2017	IR-2	IR-2.2	Pre-treatment	936,937
OR2LL-1-Outer-Ambient-R2-21Jun2017	OR-1	OR-1.2	Ambient	715,686
OR2LL-1-Outer-Pre-R4-15Jun2017	OR-1	OR-1.2	Pre-treatment	674,319
OR2LL-2-Outer-Heat-R3-21Jun2017	OR-1	OR-1.1	Heat	1,164,365
OR2LL-2-Outer-Pre-R3-15Jun2017	OR-1	OR-1.1	Pre-treatment	535,735
OR3AA-3-Outer-Ambient-R1-21Jun2017	OR-2	OR-2.2	Ambient	1,296,198
OR3AA-3-Outer-Pre-R1-15Jun2017	OR-2	OR-2.2	Pre-treatment	845,883
OR3AA-4-Outer-Heat-R1-21Jun2017	OR-2	OR-2.1	Heat	840,108
OR3AA-4-Outer-Pre-R3-15Jun2017	OR-2	OR-2.1	Pre-treatment	580,876

Table S2. Richness (S), Pielou's evenness index (J'), and Shannon's diversity index (H') of microbial genera from the coral SML metagenomes across different treatments and time periods.

Treatment	S	J'	H'
In situ (Time 0)	582 - 586	0.74 ± 0.02	4.72 ± 0.14
Heat (Time 2)	579 - 587	0.63 ± 0.004	4.04 ± 0.03
Ambient (Time 2)	580 - 585	0.62 ± 0.04	3.93 ± 0.24
Pre-treatment (Time 1)	578 - 585	0.55 ± 0.05	3.47 ± 0.32
